# The prognosis of patients with postoperative hyperglycemia after Stanford type A aortic dissection surgery and construction of prediction model for postoperative hyperglycemia

**DOI:** 10.3389/fendo.2023.1063496

**Published:** 2023-07-06

**Authors:** Yubin Chen, Tianyu Ouyang, Yue Yin, Cheng Fang, Can-e Tang, Fanyan Luo, Jingmin Luo

**Affiliations:** ^1^ Department of Cardiac Surgery, Xiangya Hospital, Central South University, Changsha, Hunan, China; ^2^ Department of Endocrinology, Xiangya Hospital, Central South University, Changsha, Hunan, China; ^3^ The Institute of Medical Science Research, Xiangya Hospital, Central South University, Changsha, Hunan, China; ^4^ National Clinical Research Center for Geriatric Disorders, Xiangya Hospital, Central South University, Changsha, Hunan, China; ^5^ Department of Cardiology, Xiangya Hospital, Central South University, Changsha, Hunan, China

**Keywords:** type A aortic dissection, postoperative hyperglycemia, acute kidney injury, in-hospital mortality, prediction model, risk factor

## Abstract

**Objective:**

The mortality of type A aortic dissection (TAAD) is extremely high. The effect of postoperative hyperglycemia (PHG) on the prognosis of TAAD surgery is unclear. This study aims to investigate the prognosis of patients with PHG after TAAD surgery and construct prediction model for PHG.

**Methods:**

Patients underwent TAAD surgery from January 2016 to December 2020 in Xiangya Hospital were collected. A total of 203 patients were included and patients were divided into non PHG group and PHG group. The occurrence of postoperative delirium, cardiac complications, spinal cord complication, cerebral complications, acute kidney injury (AKI), hepatic dysfunction, hypoxemia, and in-hospital mortality were compared between two groups. Data from MIMIC-IV database were further applied to validate the relationship between PHG and clinical outcomes. The prediction model for PHG was then constructed using Extreme Gradient Boosting (XGBoost) analysis. The predictive value of selected features was further validated using patient data from MIMIC-IV database. Finally, the 28-days survival rate of patient with PHG was analyzed using data from MIMIC-IV database.

**Results:**

There were 86 patients developed PHG. The incidences of postoperative AKI, hepatic dysfunction, and in-hospital mortality were significant higher in PHG group. The ventilation time after surgery was significant longer in PHG group. Data from MIMIC-IV database validated these results. Neutrophil, platelet, lactic acid, weight, and lymphocyte were selected as features for prediction model. The values of AUC in training and testing set were 0.8697 and 0.8286 respectively. Then, five features were applied to construct another prediction model using data from MIMIC-IV database and the value of AUC in the new model was 0.8185. Finally, 28-days survival rate of patients with PHG was significantly lower and PHG was an independent risk factor for 28-days mortality after TAAD surgery.

**Conclusion:**

PHG was significantly associated with the occurrence of AKI, hepatic dysfunction, increased ventilation time, and in-hospital mortality after TAAD surgery. The feature combination of neutrophil, platelet, lactic acid, weight, and lymphocyte could effectively predict PHG. The 28-days survival rate of patients with PHG was significantly lower. Moreover, PHG was an independent risk factor for 28-days mortality after TAAD surgery.

## Introduction

Aortic dissection is one of the most devastating cardiovascular diseases, which is characterized by the development of an intimal flap (1). The intimal flap is caused by the blood flowing into the aortic media and separates the true lumen from a false lumen (1). According to population-based studies, the annular incidence of aortic dissection ranges from 3 to 5 cases per 100,000 people (2, 3). Although considerable improvements have been made in the diagnosis, managements, and treatments of aortic dissection over the past decades, the mortality of aortic dissection is still extremely high nowadays (4). The common risk factors for aortic dissection include hypertension, Marfan syndrome, smoking, cocaine use, and trauma (5).

The Stanford classification divided aortic dissection into type A aortic dissection (TAAD, with ascending aorta involvement) and type B aortic dissection (TBAD, without ascending aorta involvement) (6). Most of patients with TBAD were treated with medicine or endovascular management, while the majority of patients with TAAD were managed surgically (7). The surgery for TAAD includes aortic repair or replacement, both of which are complicated and are associated with postoperative complications like spinal cord injury and acute kidney injury (8). These postoperative complications might affect the prognosis of surgery and even increase the risk of death (9). According to the data from International Registry of Acute Aortic Dissection (IRAD), the in-hospital mortality for patients with TAAD who treated with surgery was 27% (10). Hence, exploration of factors which are related to the postoperative complications or in-hospital mortality is needed to help surgeons to stratify the risk of patients with TAAD.

Hyperglycemia is an important sign of dysregulated homeostasis and is associated with poor outcomes of in-hospital patients (11). Patients underwent cardiac surgery are prone to development postoperative hyperglycemia (PHG) due to the systemic inflammation response during cardiac surgery, surgical stressors, release of catecholamine, increasement in catabolism, and the use of corticosteroids or inotropic agents (12). Hyperglycemia could induce endothelial dysfunction, glucose metabolism dysfunction, oxidative stress, inflammatory response, and mitochondrial dysfunction (13–15). Duncan et al. reported that PHG increased the risk of postoperative renal morbidity, postoperative neurologic morbidity, and mortality of patients underwent cardiac surgery (16). Moga et al. demonstrated that PHG was related to the poor outcome of patients underwent pediatric cardiac surgery (17). However, the relationship between PHG and the outcome of patients underwent surgery for TAAD is largely unknown and need further exploration.

In this study, the relationship between PHG and the outcome of patients underwent surgery for TAAD was analyzed using data from a single center. Then the prediction model for PHG was constructed using Extreme Gradient Boosting (XGBoost) analysis. Finally, the data extracted from the Medical Information Mart for Intensive Care (MIMIC)-IV database were used to further validate the relationship between PHG and the outcome of patients and the accuracy of prediction model for PHG. The effect of PHG on the survival of those patients was also analyzed using the follow-up data from MIMIC-IV database.

## Materials and methods

### Study population and data collection

Consecutive patients with TAAD who underwent open chest TAAD surgery for the first time in Xiangya Hospital, Central South University from January 2016 to December 2020 were enrolled. Exclusion criteria were as follows: pregnancy; diabetes mellitus; patients died during surgery or within 24 h after surgery; surgery patients with chronic kidney disease or chronic liver disease; patients with missing data. The study was conducted in accordance with the “Declaration of Helsinki” and the Ethics Committee of Xiangya Hospital, Central South University approved this study. Written informed consent was waived because of the observational, retrospective nature of this study.

Clinical data of patients were collected from the electronic medical records system of Xiangya Hospital. Preoperative variables in this study were as follows: demographic variables like age, gender, weight, smoking history, drinking history, blood pressure at admission, and chest pain duration; underlying conditions like diabetes mellitus, hypertension, cardiac surgery history, chronic kidney disease, chronic liver disease, and Marfan syndrome; laboratory tests like white blood cell count, neutrophil count, lymphocyte count, red blood cell count, platelet count, hemoglobin, serum albumin, serum total bilirubin, serum direct bilirubin, serum alanine aminotransferase (ALT), serum aspartate aminotransferase (AST), serum creatinine, blood glucose, lactic acid, and international normalized ratio (INR). Operative variables in this study were as follows: operation time, cardiopulmonary bypass time, aortic cross clamp time, and circulatory arrest time. Postoperative variables in this study were as follows: arterial oxygen tension–inspired oxygen concentration (PaO_2_–FiO_2_) ratio, laboratory tests as mentioned above, delirium, spinal cord complications (including postoperative new-onset hemiplegia, paraplegia, and paraparesis), cerebral complications (including stroke and coma), and cardiac complications (including postoperative new-onset atrial and ventricular fibrillation, sudden cardiac arrest, and low cardiac output syndrome).

### Definition

PHG was defined as the value of postoperative blood glucose higher than 10 mmol/L (180 mg/dL), which was based on the current guidelines of the American Society of Thoracic Surgeons practice that recommend maintaining the concentration of perioperative blood glucose lower than 10 mmol/L in patients underwent cardiac surgery (18, 19). In this study, postoperative blood glucose was based on the highest value of blood glucose within the first 24 h postoperatively. Diagnosis of acute kidney injury (AKI) was according to the Kidney Disease Improving Global Outcomes (KDIGO) criteria (20). Briefly, AKI was defined as a postoperative absolute increase in serum creatinine (SCr) levels of ≥ 26.4 umol/L within 48 h or ≥ 50% within 7 days or initiation of renal replacement therapy. Baseline SCr was defined as the first SCr test after admission. In this study, the urine output criteria in KDIGO criteria were not used because of the lack of urine output data. Postoperative hepatic dysfunction was defined as the postoperative value of the Model of End-Stage Liver Disease (MELD) score higher than 12. The postoperative value of the MELD score was calculated using the following standard formula: MELD score =11.2× ln (INR) +3.78× ln (serum total bilirubin [mg/dL]) +9.57× ln (SCr [mg/dL]) +6.43 (21). Any variable with a value less than 1 was assigned a value of 1 to avoid negative scores, and values exceeding 4 for creatinine were all replaced by 4. The highest value of the MELD score within the first 24 h postoperatively was used to define postoperative hepatic dysfunction. Postoperative hypoxemia was defined as the value of PaO_2_–FiO_2_ ratio lower than 300 mmHg (22). The highest value of PaO_2_–FiO_2_ ratio within the first 24 h postoperatively was used to define postoperative hypoxemia.

### Data extraction from MIMIC-IV database

MIMIC-IV database (version 2.0) is a large, longitudinal database that contains clinical data from the Beth Israel Deaconess Medical Center between 2008 to 2019. Patient identifiers were removed in this database to protect patient privacy. The first author of this article has completed the training and has credentialed access for MIMIC-IV database. The data files of MIMIC-IV database were downloaded from PhysioNet (https://physionet.org/content/mimiciv/2.0/). Then the database for data extraction was constructed based on these files using PostgreSQL software (version 14, The PostgreSQL Global Development Group). Patients diagnosed as aortic dissection and treated with open chest surgery were extracted from MIMIC-IV database. Briefly, patients whose diagnostic codes (the International Classification of Diseases editions [ICD], version 9 or 10) were 44101 (dissection of aorta, thoracic) or I7101 (dissection of thoracic aorta) were extracted. Then, patients underwent open chest surgery for aortic dissection were further extracted and duplicate patient data were deleted. According to the hadm_id (hospital admission ID), preoperative and postoperative variables as mentioned above of these patients were searched and extracted. Complications including postoperative AKI, postoperative hepatic dysfunction and postoperative hypoxemia were defined as mentioned above. And the AKI status, the MELD score, and PaO_2_–FiO_2_ ratio were extracted from views of MIMIC-IV database, respectively. After excluding patients with missing data, 92 patients were extracted from MIMIC-IV database. The survival status and death time of 92 patients were further obtained and used for survival analysis.

### Construction of prediction model for PHG

The data obtained from Xiangya Hospital were divided into the training set (70%) and the testing set (30%) randomly. The training set was applied to constructed the prediction model using the XGBoost package in R software (version 4.1.2; R Foundation for Statistical Computing, Vienna, Austria) which is a machine learning algorithm that could avoid over fitting problem of model. First of all, variables including age, gender, weight, and laboratory tests were selected as features of the prediction model. Then, the importance of features in the model was calculated using the XGBoost package in R software and the features were ordered by the importance of features. To further simplify the prediction model and improve the practicability of the prediction model, the top five features were selected and used to constructed the final prediction model for PHG. After that, the predictive value of these features was further validated using data extracted from MIMIC-IV database.

### Statistical analysis

Statistical analysis was performed using SPSS version 19 (IBM Corporation, Armonk, NY, USA). Continuous data were expressed as mean ± standard deviation (SD) and count data were expressed as frequency (percentage). Student’s *t* test was used to compare the continuous data with normal distribution between different groups and Mann–Whitney *U*-tests was applied to compare the continuous data with non-normal distribution. For count data, chi-square test was conducted to compare the difference in frequency between groups. Logistic regression was used to analyze the relationship between postoperative blood glucose and postoperative complications. The diagnostic value of the prediction model was determined using receiver operating characteristic (ROC) curve and the area under ROC curve (AUC). The Kaplan–Meier curve (log-rank test) was applied to compare the survival rate between different groups. Univariable and multivariable Cox regression was used to analyzed the association between PHG and 28-days mortality of patients. A value of *p* < 0.05 was considered to be statistically significant.

## Results

### Demographic characteristics

There were 242 patients underwent open chest TAAD surgery for the first time in Xiangya Hospital, Central South University from January 2016 to December 2020. Among them, 39 patients were excluded: three patients with pregnancy, eight patients with diabetes mellitus, seven patients died intraoperatively or within 24 h after surgery, 12 patients with chronic kidney or liver disease, 9 patients with missing data. Finally, 203 patients were included in this study.

Preoperative and operative variables of these patients were showed in **Table 1**. Out of 203 patients, there were 86 patients with PHG according to the definition above. The mean age of patients in non PHG and PHG group were 49.91 ± 10.49 and 52.43 ± 11.91, respectively. 75.21% of patients in non PHG group were male, while 65.12% of patients in PHG group were male. We noticed that the mean weight of patients in non PHG group was significantly higher than that of patients in PHG group (72.15 ± 13.29 vs. 68.33 ± 13.04, *p*=0.042). And the proportion of patients with drinking history was significantly higher in non PHG group (42.74% vs. 30.23%, *p*=0.047). In addition, the counts of white blood cell and neutrophil were significantly greater in non PHG group (12.30 ± 4.82*10^9/L vs. 10.63 ± 3.46*10^9/L, *p*=0.004; 10.19 ± 4.28*10^9/L vs. 8.64 ± 3.24*10^9/L, *p*=0.004). Surprisingly, there was no significant difference in preoperative blood glucose level between two groups. In terms of operative variables, operation time, cardiopulmonary bypass time, aortic cross clamp time, and circulatory arrest time of patients from different groups showed no significant difference.

**Table 1:** Preoperative and operative variables of patients in different groups.

Variables	non PHG group (n=117)	PHG group (n=86)	*p* value
Age (years)	49.91 ± 10.49	52.43 ± 11.91	0.112
Gender (F/M)	29/88	30/56	0.080
Weight (kg)	72.15 ± 13.29	68.33 ± 13.04	0.042
Hypertension (n (%))	90 (76.92)	58 (67.44)	0.090
Marfan syndrome (n (%))	3 (2.56)	6 (6.98)	0.123
Cardiac surgery history (n (%))	8 (6.84)	3 (3.49)	0.237
Smoking history (n (%))	62 (52.99)	44 (51.16)	0.454
Drinking history (n (%))	50 (42.74)	26 (30.23)	0.047
Systolic BP (mmHg)	134.53 ± 26.20	149.55 ± 145.04	0.274
Diastolic BP (mmHg)	67.10 ± 13.70	65.20 ± 16.95	0.377
INR	1.15 ± 0.17	1.28 ± 1.05	0.201
White blood cells (×10^9^/L)	12.30 ± 4.82	10.63 ± 3.46	0.004
Red blood cells (×10^12^/L)	4.15 ± 0.71	4.05 ± 0.58	0.266
Hemoglobin (g/L)	125.10 ± 23.84	123.67 ± 18.18	0.629
Platelet (×10^9^/L)	172.96 ± 71.50	163.70 ± 68.25	0.350
Neutrophil (×10^9^/L)	10.19 ± 4.28	8.64 ± 3.24	0.004
Lymphocyte (×10^9^/L)	1.00 ± 0.60	1.06 ± 0.56	0.463
Albumin (g/L)	37.63 ± 5.59	37.54 ± 5.34	0.906
Total bilirubin (umol/L)	19.06 ± 12.59	19.28 ± 13.67	0.905
Direct bilirubin (umol/L)	8.59 ± 5.57	9.07 ± 6.93	0.588
ALT (U/L)	60.31 ± 251.71	61.56 ± 157.19	0.968
AST (U/L)	67.39 ± 222.19	120.43 ± 467.83	0.284
Creatinine (umol/L)	126.07 ± 90.53	111.11 ± 51.46	0.138
Blood glucose (mmol/L)	8.17 ± 3.05	7.66 ± 1.84	0.175
Lactic acid (mmol/L)	1.70 ± 1.25	1.47 ± 1.28	0.195
Operation time (min)	458.50 ± 106.37	464.80 ± 114.98	0.699
Cardiopulmonary bypass time (min)	206.97 ± 65.79	211.67 ± 69.51	0.624
Aortic cross clamp time (min)	103.42 ± 48.18	110.62 ± 48.14	0.294
Circulatory arrest time (min)	29.54 ± 17.62	29.26 ± 18.90	0.913

PHG, postoperative hyperglycemia; BP, blood pressure; INR, international normalized ratio; ALT, alanine aminotransferase; AST, aspartate aminotransferase.

### Clinical outcomes of patients with PHG

**Figure 1:**
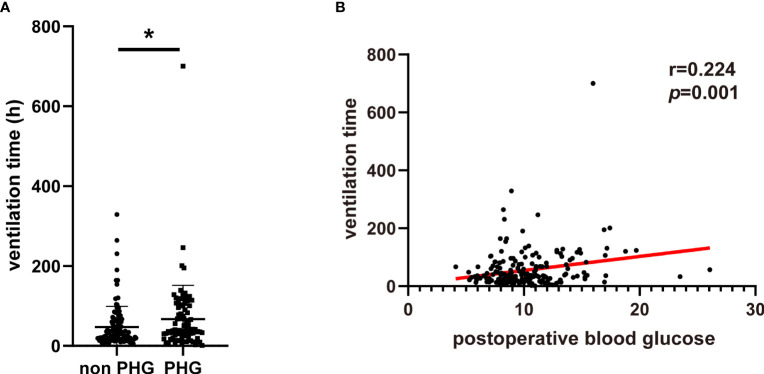
The association between PHG and ventilation. **(A)** The ventilation time of patients in different groups. **(B)** The correlation between postoperative blood glucose and ventilation time. PHG, postoperative hyperglycemia. * *p*<0.05.

The postoperative complications and in hospital mortality of patients were collected and analyzed. The results suggested that the proportions of postoperative AKI, hepatic dysfunction, and in-hospital mortality were significantly higher in PHG group compared with non PHG group (**Table 2**). The logistic analysis was further applied to investigate the association between postoperative blood glucose levels and complications. And we found that postoperative blood glucose levels were associated with a higher risk of postoperative AKI (OR: 1.144, 95% CI: 1.040-1.259, *p*=0.006) and in-hospital mortality (OR: 1.340, 95% CI: 1.150-1.562, *p*<0.001) (**Table 3**). Postoperative ventilation time of patients in PHG group was significantly greater than that of patients in non PHG group (**Figure 1A**). And the correlation analysis indicated that postoperative blood glucose levels were positively correlated with ventilation time (r=0.224, *p*=0.001) (**Figure 1B**).

**Table 2:** Postoperative complications in different groups.

Complications	non PHG group (n=117)	PHG group (n=86)	*p* value
Delirium	21 (17.95%)	12 (13.95%)	0.286
Cardiac complications	9 (7.69%)	8(9.30%)	0.435
Spinal cord complications	3 (2.56%)	3 (3.49%)	0.499
Cerebral complications	8 (6.84%)	6 (6.98%)	0.590
Postoperative acute kidney injury	38 (32.48%)	41 (47.67%)	**0.020**
Postoperative hypoxemia	103 (88.03%)	74 (86.05%)	0.415
Postoperative hepatic dysfunction	83 (70.94%)	72 (83.72%)	**0.024**
In-hospital mortality	3 (2.56%)	10 (23.26%)	**0.010**

PHG, postoperative hyperglycemia. p value in bold indicates that p value is less than 0.05.

**Table 3:** The association between postoperative blood glucose levels and complications.

Complications	postoperative blood glucose levels		
	OR	95% CI	*p* value
Delirium	0.949	0.834-1.080	0.426
Cardiac complications	1.080	0.943-1.239	0.267
Spinal cord complications	0.918	0.677-1.244	0.580
Cerebral complications	1.025	0.870-1.207	0.767
Postoperative acute kidney injury	1.144	1.040-1.259	**0.006**
Postoperative hypoxemia	0.982	0.865-1.114	0.772
Postoperative hepatic dysfunction	1.110	0.985-1.252	0.087
In hospital mortality	1.340	1.150-1.562	**<0.001**

OR, odds ratio; CI, confidence interval. p value in bold indicates that p value is less than 0.05.

### Construction and validation of prediction model for PHG

**Figure 2:**
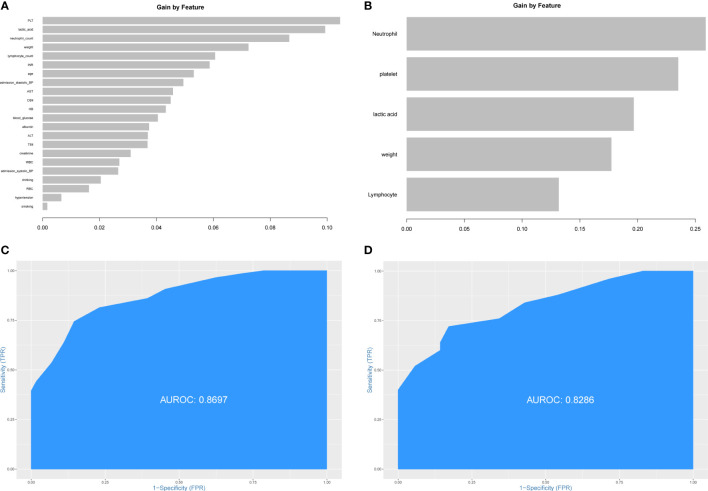
Construction of prediction model for PHG. **(A)** The importance of all preoperative variables in prediction model for feature screening. **(B)** The importance of top five features in final prediction model for PHG. **(C)** ROC curve and value of AUC of prediction model in the training set. **(D)** ROC curve and value of AUC of prediction model in the testing set. PHG, postoperative hyperglycemia; ROC curve, receiver operating characteristic curve; AUC, area under ROC curve.

The patients from Xiangya Hospital were divided into the training set (n=143) and the testing set (n=60) randomly. The construction of prediction model for PHG was based on the training set. First, all preoperative variables in **Table 1** were included to the prediction model. Then all the preoperative variables were ordered by the importance of feature which was calculated by XGBoost package in R software (**Figure 2A**). To further simplify the prediction model and improve its practicability, top five preoperative variables were selected to construct the final prediction model using data from the training set. The importance of these variables was also calculated and shown in **Figure 2B**. The diagnostic value of the prediction model was determined by ROC curve and the value of AUC in the training set was 0.8697 (**Figure 2C**). Thereafter, the data from the testing set were applied to validate the diagnostic value of the prediction model and the result demonstrated that the value of AUC in the testing set was 0.8286 (**Figure 2D**).

### Clinical outcomes of patients with PHG from MIMIC-IV database

**Figure 3:**
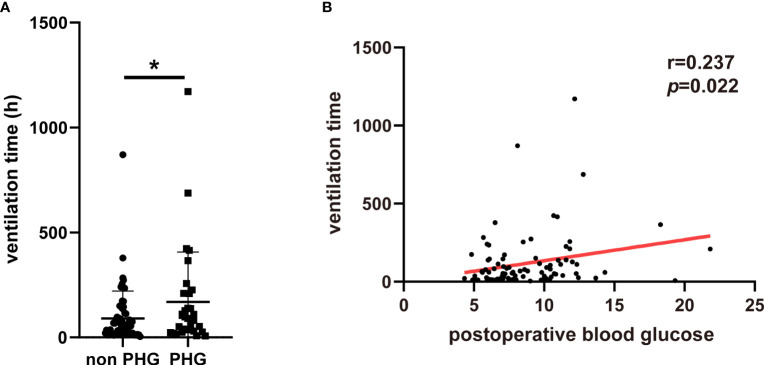
The association between PHG and ventilation using data from MIMIC-IV database. **(A)** The ventilation time of patients in different groups. **(B)** The correlation between postoperative blood glucose and ventilation time. PHG, postoperative hyperglycemia; MIMIC-IV database, the Medical Information Mart for Intensive Care IV database. * *p*<0.05.

To further explore the association between PHG and clinical outcomes of patients with TAAD, patients diagnosed as aortic dissection and treated with open chest surgery were extracted from MIMIC-IV database and divided into non PHG and PHG group according postoperative blood glucose level. After analysis, the results demonstrated that the proportions of postoperative AKI, hepatic dysfunction, and in-hospital mortality were significantly higher in PHG group compared with non PHG group (**Table 4**). The results of logistic regression indicated that postoperative blood glucose levels were associated with a higher risk of postoperative AKI (OR: 1.485, 95% CI: 1.082-2.039, *p*=0.014) (**Table 5**). The mean ventilation time of patients in PHG group was significantly greater than that of patients in non PHG group (**Figure 3A**). And the correlation analysis also revealed that postoperative blood glucose levels were positively correlated with the ventilation time (**Figure 3B**).

**Table 4:** Postoperative complications in different groups from MIMIC-IV database.

Complications	non PHG group (n=60)	PHG group (n=32)	*p* value
Postoperative acute kidney injury	46 (76.67%)	32 (1)	**0.002**
Postoperative hypoxemia	48 (80.00%)	24 (75.00%)	0.603
Postoperative hepatic dysfunction	45 (75.00%)	30 (93.75%)	**0.046**
In hospital mortality	4 (6.67%)	8(25.00%)	**0.021**

PHG, postoperative hyperglycemia; MIMIC-IV database, the Medical Information Mart for Intensive Care IV database. p value in bold indicates that p value is less than 0.05.

**Table 5:** The association between postoperative blood glucose levels and complications in data from MIMIC-IV database.

Complications	postoperative blood glucose levels		
	OR	95% CI	*p* value
Postoperative acute kidney injury	1.485	1.082-2.039	**0.014**
Postoperative hypoxemia	0.987	0.844-1.153	0.865
Postoperative hepatic dysfunction	1.246	0.985-1.578	0.067
In hospital mortality	1.177	0.994-1.392	0.058

MIMIC-IV database, the Medical Information Mart for Intensive Care IV database; OR, odds ratio; CI, confidence interval. p value in bold indicates that p value is less than 0.05.

### Difference in 28-days survival rate between non PHG and PHG groups from MIMIC-IV database

**Figure 4:**
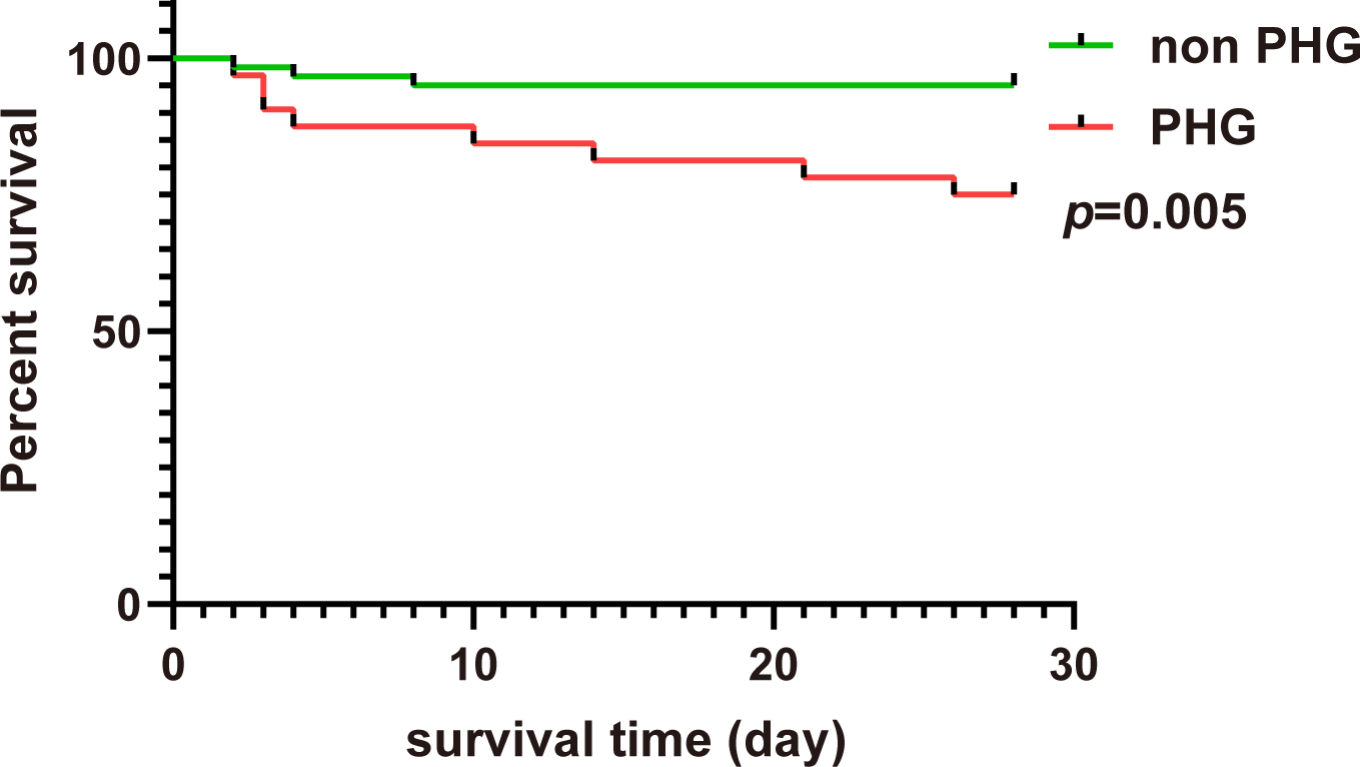
The 28-days Kaplan–Meier curve of patients in non PHG and PHG groups. PHG: postoperative hyperglycemia.

The Kaplan–Meier survival curve showed that the 28-days cumulative survival rate of patients with PHG was significantly lower than that of patients without PHG (log-rank Chi square = 7.869, *p*=0.005) (**Figure 4**). The results of univariable Cox regression revealed that PHG was a risk factor for 28-days mortality of patients with TAAD who underwent open chest surgery (**Table 6**). After adjusting variables including age, gender, and weight, PHG was also associated with a higher risk of 28-days mortality of patients (**Table 6**). Moreover, in multivariable Model 2 which further adjusted variables including age, gender, weight, postoperative AKI, postoperative hepatic dysfunction, and postoperative hypoxemia, PHG was still a significant risk factor for 28-days mortality of patients (**Table 6**).

**Table 6:** Univariable and multivariable Cox regression analysis for association between 28-days mortality and PHG using data from MIMIC-IV database.

Variables	Univariable Model			Multivariable Model 1^*^			Multivariable Model 2^#^		
	HR	95%CI	*p* value	HR	95%CI	*p* value	HR	95%CI	*p* value
PHG	5.404	1.433-20.381	**0.013**	6.386	1.650-24.716	**0.007**	6.781	1.395-32.957	**0.018**

PHG: postoperative hyperglycemia; MIMIC-IV database: the Medical Information Mart for Intensive Care IV database; HR: hazard ratio; CI: confidence interval.

*: Adjusted variables for the multivariable model 1: age, gender, and weight.

#: Adjusted variables for the multivariable model 2: age, gender, weight, postoperative AKI, postoperative hepatic dysfunction, and postoperative hypoxemia. p value in bold indicates that p value is less than 0.05.

### Construction of the prediction model for PHG using data from MIMIC-IV database

**Figure 5:**
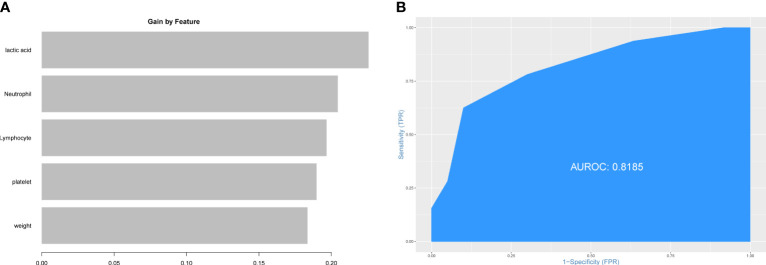
Construction of prediction model for PHG using data from MIMIC-IV database. **(A)** The importance of features in prediction model. **(B)** ROC curve and value of AUC of prediction model. PHG, postoperative hyperglycemia; MIMIC-IV database, the Medical Information Mart for Intensive Care IV database.

First, we tried to validate the prediction model for PHG above using patient data from MIMIC-IV database. However, the value of AUC was low (0.5245) (**Supplementary Figure 1**) which meant the model above did not have diagnostic value for PHG in patients from MIMIC-IV database. We speculated that the difference in race or laboratory instrument might limit the use of the model above. Then, five features mentioned above were applied to construct predictive model using patient data from MIMIC-IV database. The importance of feature was showed in **Figure 5A** and the value of AUC of this model in patient data from MIMIC-IV database was 0.8185 (**Figure 5B**).

## Discussion

Aortic dissection is considered as a complex and life-threatening cardiovascular disease. According to hospital-based studies, the incidence of aortic dissection is 3-5 cases per 100000 people per year (2, 3). However, these studies did not take pre-admission deaths into account, which would underestimate the incidence of aortic dissection (23). There is a population-based prospective study that reported a higher incidence of aortic dissection, which is 15 cases per 100000 people per year (24). The increased aortic wall stress and compromised medial layer of aortic wall are two key pathologies of aortic dissection (25). Inflammation and extracellular matrix degradation are main mechanisms that contribute to the onset of aortic dissection, but the exact trigger of aortic dissection is not clear (1). When aortic dissection occurred, the formation of false lumen and abnormal blood flow would lead to organ malperfusion, tamponade, or even aortic rupture (1). Considering the poor outcomes of aortic dissection, rapid diagnosis and treatment are needed to improve the survival of patients with aortic dissection (26). The main treatment for patients with TAAD is surgery, which includes aortic root replacement, supracoronary replacement of the ascending aorta, aortic valve-sparing aortic root repair, and frozen elephant trunk repair (8). Although significant improvement has been made in the diagnosis, decision making, and treatment of patients with TAAD, the mortality rate of these patients is still high. According to the data derived from IRAD, the in-hospital mortality rate of patients with TAAD was 22% and the surgical mortality rate was 18% (27). Therefore, it is necessary to explore risk factor which is associated with postoperative complications and in-hospital mortality to better predict the prognosis of patients with TAAD.

Hyperglycemia is related with poor outcomes of hospitalized patients and usually occurs during the perioperative period, which indicates a higher risk for mortality and morbidity of patients (11, 28). Duncan et al. reported that increasements in intraoperative and postoperative blood glucose levels could predict risk for mortality and morbidity of patients underwent cardiac surgery (16). Moga et al. found that hyperglycemia after pediatric cardiac surgery was associated with increased risk of postoperative hepatic insufficiency, stroke, and prolonged ventilation time (17). Lin et al. indicated that there was a strong correlation between admission hyperglycemia and postoperative mechanical ventilation time (29). However, there is few studies that focus on the relationship between PHG and outcomes of patients with TAAD who underwent open chest surgery. In this study, patient data were collected and extracted from our hospital and MIMIC-IV database to investigate the relationship. First of all, an appropriate threshold for PHG is needed. Current guidelines of the American Society of Thoracic Surgeons practice recommend maintaining the concentration of perioperative blood glucose lower than 180 mg/dL (10 mmol/L) in patients underwent cardiac surgery (18, 19). In the studies of Greco et al. (30), Subramaniam et al. (31), Matsumoto et al. (32), Kim et al. (33), and Moga et al. (17), the thresholds of intraoperative and postoperative hyperglycemia were 180 mg/dL (10 mmol/L). Therefore, we applied the same threshold to define PHG in this study. There were 203 patients included in this study and 86 (42.36%) of these patients exhibited PHG. The data of Greco et al. (30) and Moga et al. (17) demonstrated that the proportions of PHG in adult patients and children who underwent cardiac surgery were 36% and 21.50% respectively, both of which used the same threshold for PHG. The proportion of PHG in patients underwent surgery for TAAD was higher than patients underwent cardiac surgery. PHG is a stress-induced phenomenon (13) and the complicated surgical procedures and longer cardiopulmonary bypass time are both strong stressors which might increase the incidence of PHG. Most of patients with TAAD were bedridden and admitted through emergency department and the height of some patients was not recorded, so the body mass index was not available in this study. The mean weight of patients in PHG group was significantly lower than that of patients in non PHG group. In the study of Moga et al., although not reaching statistical significance, the mean weight of children with PHG were lower than that of children without PHG (17). We also noticed that the counts of white blood cell and neutrophil of patients in PHG group were significantly lower than patients in non PHG group. However, there is no other study reports similar results and the role of preoperative counts of white blood cell and neutrophil in PHG needs further exploration. In terms of operative variables, the operation time, cardiopulmonary bypass time, and aortic cross clamp time of patients in PHG group were slightly longer than patients in non PHG group, but did not reach statistical significance. Duncan et al. showed that the aortic cross clamp time of patients developed severe PHG (blood glucose higher than 200 mg/dL) after cardiac surgery was significantly longer than that of patients who did not develop severe PHG (16). The difference might be due to the relatively small sample size of our study and the different threshold for PHG.

AKI is a common complication of surgery for TAAD and is associated with the increased risk of mortality (34). The diagnosis of AKI was according to the KDIGO criteria which aims to diagnose AKI at early stage (20), and the incidence of AKI in this study was 38.92%. The results of logistic regression indicated that the concentration of postoperative blood glucose was associated with a higher risk of AKI. And data from MIMIC-IV database further validated that the concentration of postoperative blood glucose was a risk factor for AKI. Song et al. reported that intraoperative hyperglycemia was related to the renal dysfunction after off-pump coronary artery bypass (35). And Duncan et al. demonstrated that severe PHG was associated with the onset of renal morbidity in patients after cardiac surgery (16). Researches revealed that dysregulation of blood glucose induced AKI by oxidative stress, inflammation, and endothelial dysfunction (36). The overall in-hospital mortality rate in this study was 6.40%, which is slightly lower than other studies focused on TAAD (37, 38). The explanation for the lower in-hospital mortality rate is that due to the purpose of this study, patients with chronic kidney and liver diseases and patients died intraoperative or within 24 h after surgery were excluded. Using data from our hospital, we found that the concentration of postoperative blood glucose was associated with a higher risk of in-hospital mortality. Data extracted from MIMIC-IV database also indicated that the concentration of postoperative blood glucose might be a risk factor for in-hospital mortality of patients with TAAD, but not reaching statistical significance. It was reported by Duncan et al. that severe PHG was a significant risk factor for in-hospital mortality (16). Lin et al. suggested that admission blood glucose level was not associated with in-hospital mortality of patients underwent surgery for TAAD (29). However, there is few studies explore the relationship between PHG and in-hospital mortality of patients under surgery for TAAD. To further elucidate the effect of PHG on the survival rate of patients under surgery for TAAD, we extracted the follow-up data of patients under surgery for TAAD from MIMIC-IV database. The results of Kaplan–Meier curve (log-rank test) demonstrated that 28-days survival rate of patients with PHG were significantly lower than patients without PHG. And the Cox regression analysis revealed that PHG was an independent risk factor for 28-days mortality of patients under surgery for TAAD, even after adjusting demographic and postoperative variables including age, gender, weight, postoperative AKI, postoperative hepatic dysfunction, and postoperative hypoxemia. The influence of PHG in short term survival of patients with TAAD needs further validation using data from multi-centers.

Considering the importance of PHG in the prognosis of patients with TAAD, we constructed prediction model for PHG using data from our hospital *via* XGBoost analysis, a machine learning algorithm which could effectively avoid overfitting (39). After screening, neutrophil count, platelet count, concentration of lactic acid, weight, and lymphocyte count were selected as features to construct the model. The values of AUC in training set and testing set of our hospital were 0.8697 and 0.8286 respectively. However, when the model was applied to the data from MIMIC-IV database, the value of AUC was merely 0.5245, which reflected a poor diagnostic value. We speculated that the difference in race, laboratory instruments, or other confounding factors would affect the use of this model in other data. Therefore, five features mentioned above were applied to construct another prediction model using data from MIMIC-IV database and the new model exhibited satisfying diagnostic value with the value of AUC = 0.8185. These results indicated that the combination of the five features could be used to predict PHG in patients with TAAD but the model needs to be modified in certain circumstance like patients with different races. And how these features affect PHG needs further exploration.

There are several limitations in this study. First, this is a single center retrospective study. But the data from MIMIC-IV database were applied to validate the findings and model in this study, which could reduce this weakness to some extent. Second, due to the lack of urine output data, the urine output criteria in KDIGO criteria were not used in this study, and only the SCr criteria were used. In studies of Wang et al. (40) and Li et al. (37), only SCr criteria were applied to diagnose AKI, too. Third, patients with diabetes mellitus were excluded and postoperative blood glucose was based on the highest value of blood glucose within the first 24 h postoperatively. So, the use of insulin was not taken into consideration in this study, which might be a confounding factor and need further exploration in next study.

## Conclusion

PHG was significantly associated with the occurrence of AKI, hepatic dysfunction, increased ventilation time, and in-hospital mortality after TAAD surgical repair. The feature combination of neutrophil count, platelet count, concentration of lactic acid, weight, and lymphocyte count could effectively predict PHG of patients with different races. The 28-days survival rate of patients with PHG was significantly lower than patients without PHG. Moreover, PHG was an independent risk factor for 28-days mortality of patients after TAAD surgical repair.

## Data availability statement

The original contributions presented in the study are included in the article/**Supplementary Materials**. Further inquiries can be directed to the corresponding authors.

## Ethics statement

The study was conducted in accordance with the “Declaration of Helsinki” and the Ethics Committee of Xiangya Hospital, Central South University approved this study (No.201911487). Written informed consent was waived because of the observational, retrospective nature of this study.

## Author contributions

YC extract data from MIMIC-IV database, analyzed the data, completed figures, and wrote manuscript. TO, YY, and CF collected data from our hospital, processed the raw data, and completed tables. C-ET, JL, and FL designed the research. JL and FL reviewed and edited the manuscript. All authors contributed to the article and approved the submitted version.
