# Supplementary figures and images for: The prognosis of patients with postoperative hyperglycemia after Stanford type A aortic dissection surgery and construction of prediction model for postoperative hyperglycemia
Source: Front Endocrinol (Lausanne). 2023 Jul 6;14:1063496. doi: 10.3389/fendo.2023.1063496 (PMC10357292; doi:10.3389/fendo.2023.1063496)

ROC Curve

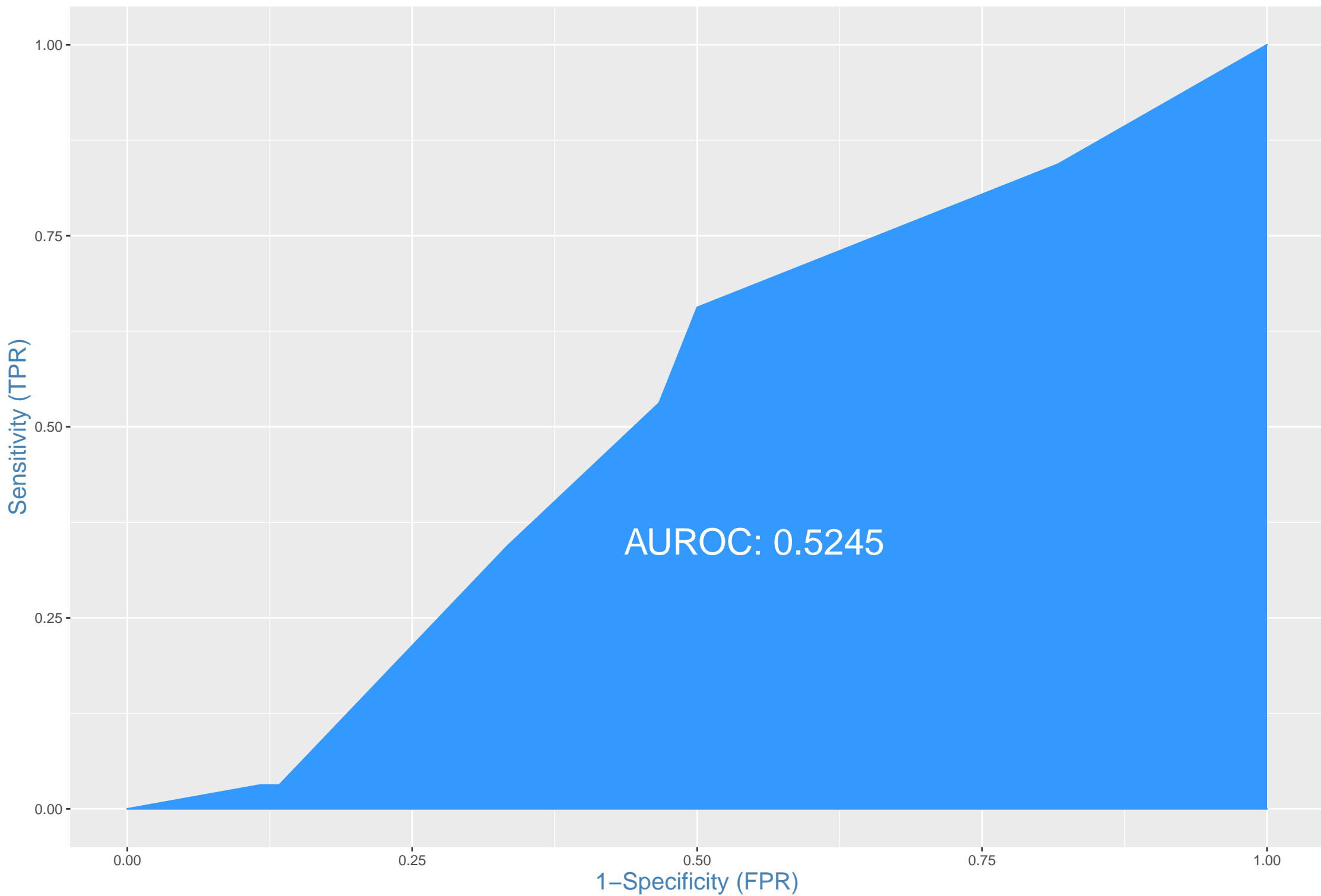

Supplement: Supplementary file 1 [file DataSheet_1.pdf]
